# Short-term clinical and radiographic outcomes of a 3D-printed trabecular titanium acetabular cup in primary total hip arthroplasty for Crowe type I–III developmental dysplasia of the hip with limited acetabular bone defects: a retrospective comparative study

**DOI:** 10.3389/fmed.2026.1906028

**Published:** 2026-08-31

**Authors:** Chengxiao Li, Lilei Song, Junheng Zhang, Bo Liu, Zhaolong Wang, Yongqiang Fan, Binquan Zhang, Yiding Liu, Kun Xiang, Yifan Niu, Guangyang Lu, Huijie Li

**Affiliations:** 1 Department of Orthopaedics, The Third Hospital of Hebei Medical University, Shijiazhuang, China; 2 Hebei Cangzhou Hospital of Integrated Traditional Chinese and Western Medicine, Cangzhou, China

**Keywords:** 3D-printed trabecular titanium acetabular cup, developmental dysplasia of the hip, total hip arthroplasty, porous titanium acetabular cup, radiographic outcomes, osseointegration

## Abstract

**Objective:**

To compare the short-term clinical and radiographic outcomes of a 3D-printed trabecular titanium acetabular cup system (AK Medical, Beijing, China) with a conventional porous titanium-coated acetabular cup system (Pinnacle Gription Sector Acetabular System, DePuy Synthes/Johnson & Johnson, USA) in primary total hip arthroplasty (THA) for Crowe type I–III developmental dysplasia of the hip (DDH) with limited acetabular bone defects not requiring metal augments or structural bone grafting.

**Methods:**

This retrospective study included 104 patients (105 hips) who underwent primary THA for DDH between January 2022 and July 2023, including 51 hips in the 3D-printed trabecular titanium cup group and 54 hips in the conventional porous titanium-coated cup group, with a mean follow-up of approximately 24 months. The primary outcome was the proportion of cups meeting at least 3 of the 5 Moore radiographic criteria at final follow-up, with early revision classified as failure of the index cup. VAS and HHS were analyzed using patient-clustered generalized estimating equations (GEE). The primary radiographic endpoint was compared using Fisher's exact test, with stabilized inverse probability of treatment weighting (IPTW) used as an adjusted sensitivity analysis.

**Results:**

Operative time, intraoperative blood loss, length of stay, and postoperative acetabular cup position parameters were similar between groups. VAS scores were lower in the 3D-printed cup group at 1 week, 1 month, and 3 months postoperatively. A significant group-by-time interaction was observed for HHS; HHS was higher in the 3D-printed group at 1 and 6 months, the between-group difference at 3 months did not reach statistical significance, and outcomes were similar between groups from 12 months onward. In the primary analysis, which conservatively classified early revision as failure, the proportions meeting the prespecified final-follow-up radiographic criterion were 46/51 (90.2%) and 40/54 (74.1%), respectively (*P* = 0.042; crude OR = 3.22, 95% CI: 1.07–9.73). After stabilized IPTW, the weighted proportions were 92.9% and 75.7%, with a weighted OR of 4.21 (95% CI: 1.35–13.17, *P* = 0.013). In the serial-radiograph subset of 82 hips, the proportions meeting the ≥3/5 Moore criterion at 3 and 6 months were similar between groups and increased over time; *P* for the group-by-time interaction was 0.097. Complication events were infrequent, and safety findings were interpreted descriptively.

**Conclusion:**

In primary THA for Crowe type I–III DDH with limited acetabular bone defects not requiring augments or structural bone grafting, the 3D-printed trabecular titanium acetabular cup system was associated with a higher proportion of hips meeting the final-follow-up radiographic integration endpoint and with better early VAS and HHS outcomes. No clear early between-group difference in the radiographic endpoint was observed at 3 or 6 months. This study compared two specific implant systems; longer follow-up is required to evaluate long-term fixation and implant survivorship.

## 1 Introduction

Developmental dysplasia of the hip (DDH) encompasses a spectrum of congenital abnormalities, including acetabular and femoral dysplasia, hip instability, subluxation, and dislocation, with a global neonatal incidence of approximately 1–3 per 1,000 live births (1). DDH alters joint biomechanics, increases articular cartilage loading, and accelerates osteoarthritis progression, and is a major cause of secondary hip degeneration in adults younger than 40 years (2–4). Worldwide, untreated or inadequately treated DDH may lead to pain, functional limitation, and impaired quality of life, and total hip arthroplasty (THA) is often the definitive treatment for restoring mobility and relieving symptoms (3, 5). Acetabular components in THA have evolved from cemented fixation toward cementless porous-coated implants designed to promote osseointegration and long-term stability (3, 6). Traditional porous titanium coatings, such as sintered beads or plasma-sprayed hydroxyapatite coatings, have improved initial fixation and bone ingrowth, with reported 10–15-year survivorship exceeding 90% in primary THA (5, 6). However, patients with DDH frequently have a shallow acetabulum, anterosuperior bone deficiency, and excessive femoral anteversion, which complicate reconstruction and increase the risks of instability and revision (7). More recently, highly porous structures have been developed to mimic trabecular bone and promote vascularization, osteoconduction, and load transfer, particularly in complex cases with bone defects (6, 8).

Despite advances in acetabular cup design, conventional porous titanium cups have several limitations in DDH, including inadequate primary stability in osteoporotic or bone-deficient host bone, coating delamination, and suboptimal osseointegration; these factors may contribute to a higher revision risk than that observed in patients with osteoarthritis (odds ratio, 1.66) (6, 9). Such cups generally have relatively lower porosity (e.g., 40%–60%) and smaller pore sizes (250–400 μm), which may restrict bone ingrowth and contribute to stress shielding, whereby stiffness mismatch leads to periprosthetic bone resorption and aseptic loosening (6). In Crowe type I–III DDH, inadequate acetabular coverage and poor bone quality may necessitate extensive bone grafting or supplemental reconstruction with conventional implants, increasing surgical complexity and the risks of graft resorption or nonunion (7, 10).

Three-dimensional (3D) printing has been increasingly applied in medicine, particularly in orthopaedics, to address limitations of conventional manufacturing. Traditional acetabular components are commonly produced using subtractive manufacturing or coating-based additive processes. Modern 3D printing enables true additive manufacturing and the creation of controlled porous architectures that are difficult to achieve using conventional subtractive or coating techniques (11–13). Electron-beam melting can melt titanium at temperatures approaching 2,000 °C to manufacture acetabular cups with specifically designed and controllable porous surface structures. Such designs can reduce the elastic modulus of the cup and more closely approximate the mechanical characteristics of trabecular bone (6, 8). Emerging 3D-printed trabecular titanium cups feature higher porosity (60%–90%), larger pore sizes (600–1,000 μm), and an elastic modulus closer to cancellous bone (0.5–1.3 GPa), potentially increasing friction, promoting bone ingrowth and load distribution, and reducing stress shielding (6, 8, 14). Recent studies have reported favorable mid-term osseointegration and functional recovery in revision THA; however, evidence for their use in primary THA for DDH remains limited, while conventional cups continue to face challenges related to early stability and long-term survivorship (6, 14).

Against this background, this study aimed to compare the short-term clinical and radiographic outcomes of a 3D-printed trabecular titanium acetabular cup system (AK Medical, Beijing, China) and a conventional porous titanium-coated acetabular cup system (Pinnacle Gription Sector Acetabular System, DePuy Synthes/Johnson & Johnson, USA) in primary THA for Crowe type I–III DDH with limited acetabular bone defects not requiring metal augments or structural bone grafting. The study focused on early pain relief, functional recovery, perioperative complications, acetabular cup position, host-bone coverage, and radiographic signs suggestive of osseointegration. Given the retrospective, nonrandomized design, the primary objective was to evaluate associations between the two specific acetabular cup systems and short-term outcomes rather than to establish causal superiority or general material-level superiority.

## 2 Patients and methods

### 2.1 Study population

This retrospective cohort study was conducted by reviewing the medical records of patients who underwent primary THA for osteoarthritis secondary to DDH at The Third Hospital of Hebei Medical University between January 2022 and July 2023. According to the inclusion and exclusion criteria, 104 patients (105 hips) were ultimately included. Hips were grouped according to the acetabular cup system implanted: 51 hips received the 3D-printed cup and 54 received the conventional cup. One patient underwent staged bilateral THA, with a different acetabular cup system implanted in each hip; therefore, the two hips were assigned to different treatment groups. Both acetabular cup systems were clinically available and used concurrently during the study period. Implant selection was part of routine clinical decision-making. Preoperative radiographs and intraoperative findings were first used to determine whether a hemispherical press-fit acetabular cup could provide sufficient host-bone coverage and primary stability without metal augments, reinforcement rings, cages, or structural femoral-head bone grafting. Thus, acetabular morphology and bone quality were used primarily to determine suitability for the reconstruction strategy evaluated in this study. When both cup systems were considered technically suitable, the surgeon discussed their relevant characteristics with the patient, including the porous architecture and theoretical fixation advantages of the 3D-printed cup, the established clinical use of the conventional cup, the uncertainty regarding clinical superiority between the systems, and differences in implant-related costs. The final choice was made through shared decision-making, generally incorporating patient preference and individual financial considerations. No fixed rule assigned a particular acetabular cup system on the basis of Crowe classification, Hartofilakidis classification, osteoporosis status, or any other single preoperative factor. Standard preoperative pelvic radiographs and clinical assessment were used for surgical planning; CT was not routinely required, and no recorded CT-based implant-selection algorithm was used. All procedures were performed through a posterolateral approach by the same senior surgeon, and the distribution of surgery year was compared between groups to assess potential temporal and learning-curve effects.

The study was approved by the Ethics Committee of The Third Hospital of Hebei Medical University (approval no. K-2003-013-1). The requirement for written informed consent was waived because of the retrospective study design. Only aggregated, nonidentifiable clinical and radiographic information is reported. All procedures involving human participants were conducted in accordance with the principles of the Declaration of Helsinki as revised in 2013.

### 2.2 Inclusion and exclusion criteria

The inclusion criteria were as follows: (1) DDH of Crowe type I, II, or III according to the classification described by Crowe et al. (15), with typical clinical and radiographic features of DDH in the operated hip; (2) primary THA performed through a posterolateral approach; (3) implantation of either a 3D-printed trabecular titanium acetabular cup or a conventional porous titanium-coated acetabular cup; and (4) a minimum follow-up of 18 months with complete and available clinical and radiographic follow-up data.

The exclusion criteria were as follows: (1) history of hip infection; (2) previous hip surgery; (3) follow-up of <18 months; (4) incomplete follow-up data; (5) marked developmental deformity or abnormality on the femoral side; (6) disorders that could substantially impair postoperative rehabilitation, such as poliomyelitis or Parkinson disease; (7) severe osteoporosis; and (8) acetabular bone coverage or primary stability so poor that reconstruction required a metal augment, reinforcement ring, cage, or structural autologous femoral-head bone graft.

“Limited acetabular bone defects” was used to define the scope of acetabular reconstruction in this study and referred to cases in which reconstruction could be completed with a hemispherical press-fit acetabular cup and intraoperative primary stability could be achieved without a metal augment, reinforcement ring, cage, or structural autologous femoral-head bone graft. All included hips met these reconstruction criteria.

Preoperative DDH anatomy was characterized using the Crowe and Hartofilakidis classifications. Hartofilakidis classification was determined from standard preoperative anteroposterior pelvic radiographs: type A was acetabular dysplasia in which the femoral head remained within the original true acetabulum; type B was low dislocation in which the femoral head articulated with a false acetabulum whose inferior margin contacted or partially overlapped the superior margin of the true acetabulum; and type C was high dislocation in which the femoral head was displaced superoposteriorly with no contact between the true and false acetabula (16). The Crowe classification was used to describe the degree of proximal femoral-head displacement, whereas the Hartofilakidis classification further characterized the anatomical relationship between the femoral head and the true and false acetabula. Preoperative CE and Tönnis angles were used as continuous radiographic measures of acetabular coverage and acetabular roof inclination, respectively.

Two assessors independently assigned the main Hartofilakidis classification using standard preoperative anteroposterior pelvic radiographs. Each assessor recorded type A, B, or C, and interobserver agreement was evaluated using Cohen's *κ* with a 95% confidence interval (CI). For cases with discrepant primary classifications, the two assessors jointly reviewed the anatomical relationship of the femoral head to the true and false acetabula and reached a final consensus classification; the consensus result was used in subsequent baseline comparisons and adjusted analyses. B1/B2 and C1/C2 subtypes were retained in the radiographic reading records but were not included in the primary statistical analysis.

### 2.3 Acetabular implants

**Figure 1:**
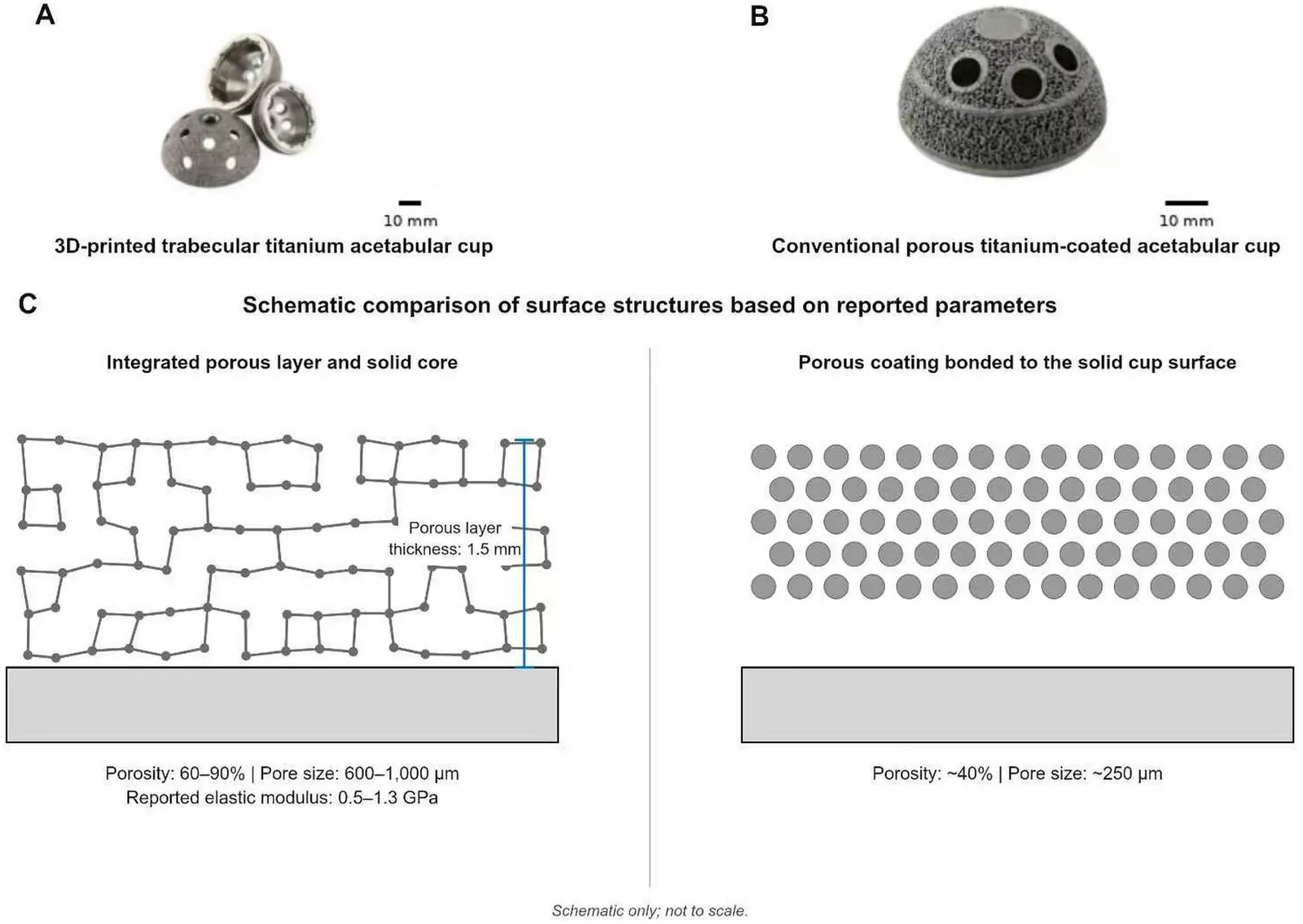
Comparison of the acetabular cup systems evaluated in this study. **(A)** Image of the 3D-printed trabecular titanium acetabular cup (AK Medical, Beijing, China). **(B)** Image of the conventional porous titanium-coated acetabular cup (Pinnacle Gription Sector Acetabular System, DePuy Synthes/Johnson & Johnson, USA). Scale bars in panels A and B are 10 mm. **(C)** Schematic comparison of reported surface structures and characteristics. Panel C is illustrative, is not drawn to scale, and is not intended for quantitative microstructural comparison.

The 3D-printed group received a 3D-printed trabecular titanium acetabular cup (AK Medical, Beijing, China) (Figure 1). Because the solid metal shell and surface porous layer are manufactured as a continuous integrated structure, the implant is theoretically more resistant to coating delamination and corrosion. The outer surface contains a 1.5-mm-thick trabecular porous titanium structure with a mean porosity of 60%–90%, a pore size of 600–1,000 μm, and interconnected pores. Its mean elastic modulus is 0.5–1.3 GPa, approximating that of human cancellous bone; the friction coefficients between the cup and cancellous and cortical bone are 1.08 and 0.93, respectively. The conventional group received a conventional porous titanium-coated acetabular cup (Pinnacle Gription Sector Acetabular System, DePuy Synthes/Johnson & Johnson, USA). This implant has a sintered titanium-bead porous coating, with a mean porosity of approximately 40% and a pore size of approximately 250 μm. Figure 1 presents side-by-side product images and an illustrative schematic comparison based on reported surface parameters.

### 2.4 Surgical technique

All THAs were performed through a posterolateral approach by the same senior surgeon. General anesthesia or continuous epidural anesthesia combined with a nerve block was used. After satisfactory anesthesia, the patient was placed in the lateral decubitus position and the hip was exposed through a posterolateral approach. The femoral head was resected, and the surrounding capsule, synovium, soft tissue within the true acetabular fossa, and other obstructing tissues were removed to fully expose the acetabulum. After identification of the true acetabulum, the hip center of rotation was reconstructed anatomically or moderately elevated according to the acetabular defect to improve cup coverage. The acetabulum was sequentially reamed to an appropriate size. A trial component was inserted and adjusted until satisfactory position and subjective primary stability were achieved, after which the final acetabular component was implanted. The use of supplemental screws was determined by the surgeon according to intraoperative press-fit stability and host-bone coverage; when supplemental fixation was required, two screws were used. Archived operative records did not contain quantitative insertion torque, interface micromotion, or a prespecified standardized threshold. The liner was then inserted. All included hips were judged to have achieved primary stability. An appropriate femoral stem and femoral head were selected according to proximal femoral morphology to complete hip reconstruction. Implant position was confirmed fluoroscopically before wound closure.

### 2.5 Perioperative management

A first-generation cephalosporin was administered 30–60 min before skin incision and during the first 24 h postoperatively for infection prophylaxis. After recovery from anesthesia, all patients began active exercises in bed. Full weight bearing was generally permitted from postoperative day 1; patients with an intraoperative femoral fracture were allowed to begin weight bearing at 4 weeks postoperatively. Low-molecular-weight heparin (LMWH) and analgesics were routinely administered for prevention of deep vein thrombosis (DVT) and pain control, respectively. Skin sutures were removed at approximately 14 days postoperatively according to wound healing.

### 2.6 Clinical and radiographic assessment

Routine clinical and/or radiographic follow-up was scheduled preoperatively, at 1 week, 1 month, 3 months, 6 months, and 1 year postoperatively, and every 6 months thereafter. For patients living far from the study center or unwilling to return for follow-up, clinical outcomes were assessed by telephone and radiographs obtained at local hospitals were transmitted electronically for radiographic evaluation. Clinical and radiographic outcomes were assessed by two attending orthopaedic surgeons who had not participated in the index procedures. During radiographic review, only the radiographs were provided; VAS, HHS, complications, revision status, and other clinical outcomes were withheld, and the assessors remained blinded to clinical outcomes. The radiographs were not labeled by treatment group. Because structural differences between the two acetabular cups might be recognizable on radiographs, implant type could nevertheless have been identified.

Clinical assessments included the visual analog scale (VAS), Harris Hip Score (HHS), and perioperative variables. Perioperative variables included operative time, intraoperative blood loss, length of stay, and intraoperative and postoperative complications, including wound-healing complications, nerve injury, DVT, dislocation, periprosthetic joint infection, periprosthetic fracture, early aseptic loosening, and revision. Pain was assessed on a 0–10 VAS, with higher scores indicating greater pain. The HHS was used to assess hip function on a 100-point scale encompassing pain, function, deformity, and range of motion (17).

Radiographic assessment included acetabular cup inclination, anteversion, leg-length discrepancy (LLD), hip center of rotation (COR), cup center-edge (Cup-CE) angle, bone coverage index (BCI), and radiographic signs suggestive of acetabular cup osseointegration. Cup inclination was defined as the angle between the interteardrop line and the tangent to the acetabular cup. Cup anteversion was measured using the method of Lewinnek et al. (18). The vertical center of rotation (V-COR) was defined as the perpendicular distance from the femoral-head center to the interteardrop line; the horizontal center of rotation (H-COR) was defined as the horizontal distance from the femoral-head center to a vertical line through the lowest point of the teardrop. The Cup-CE angle was defined as the angle between the line connecting the hip center to the lateral edge of host bone and the perpendicular to the interteardrop line (19). BCI was defined as the ratio of the horizontal width of the cup covered by host bone to the total horizontal width of the cup (19).

The DeLee and Charnley zonal system was used to assess cup position based on the width of radiolucent lines (RLLs), change in cup inclination, and migration distance (20, 21). Cup stability was defined as the absence of progressively widening RLLs, a change in inclination of no more than 10°, and migration of no more than 6 mm. Radiographic signs suggestive of osseointegration were evaluated according to the criteria described by Moore et al. (22). A cup was considered to meet the radiographic criterion suggestive of osseointegration when at least 3 of the following 5 signs were present: (1) absence of radiolucent lines; (2) superolateral buttress/sclerosis; (3) medial stress shielding; (4) radial trabeculae; and (5) inferomedial buttress/sclerosis. In the original study, this composite criterion demonstrated high positive predictive value and sensitivity for acetabular bone ingrowth (22). These findings are indirect signs on plain radiographs and should not be equated with histologically confirmed bone ingrowth or direct fixation strength. Digital tomosynthesis with metal artifact reduction and radiostereometric analysis may provide additional assessment of implant integration or migration, but these examinations were not available in this retrospective cohort (23, 24). Analyses of individual Moore signs, the total number of signs, and composite endpoints in the full cohort used the archived readings of Assessor 2; a random sample of 30 hips was independently reassessed by both assessors solely for interobserver reliability analysis. Reproducibility of continuous radiographic measurements was evaluated in a random sample of 20 hips, with both intraobserver and interobserver intraclass correlation coefficients (ICCs) >0.90. For interobserver reliability of the Moore radiographic signs, 30 hips were randomly selected and the 5 Moore signs and composite endpoints were independently assessed by both readers. Cohen's *κ* with 95% CI was used for individual Moore signs, the ≥3/5 composite endpoint, and the strict non-RLL binary endpoint (≥3/4); the total number of Moore signs was evaluated using quadratically weighted Cohen's *κ*. Observed agreement was reported for signs with no variation within the sample.

### 2.7 Outcome measures

The primary outcome was the proportion of acetabular cups meeting the radiographic criterion suggestive of osseointegration at final follow-up, defined as at least 3 of the 5 Moore signs. Secondary outcomes included perioperative measures, VAS and HHS at different postoperative time points, and radiographic parameters related to acetabular cup position. One patient in the 3D-printed group developed early aseptic loosening of the acetabular cup with dislocation on postoperative day 2 and underwent revision surgery. This case was retained as an early failure event. In the primary analysis, the index acetabular cup was classified as not meeting the ≥3/5 Moore radiographic endpoint; a sensitivity analysis assessed robustness by excluding this revision case. Because no evaluable final-follow-up radiograph of the index cup was available before revision, Moore signs observed in the revision construct were not used to determine individual radiographic signs for the index cup; therefore, this case was treated as nonevaluable in the descriptive analyses of individual Moore signs and the distribution of non-RLL sign counts at final follow-up. Because all other index cups with evaluable final radiographs had no radiolucent lines, this item was removed and the ordered count of the remaining 4 signs was analyzed. A strict sensitivity endpoint was defined as at least 3 of the 4 non-RLL signs; for this strict composite endpoint, the early revision remained classified as failure of the index cup. For the patient revised on postoperative day 2, the index cup was classified as failure of the composite endpoint at 3 months, 6 months, and final follow-up, and Moore signs from the revision construct were not used to reclassify the index-cup outcome.

### 2.8 Statistical analysis

All statistical analyses were performed using SPSS version 27.0 (SPSS Inc., Chicago, IL, USA) and Python (statsmodels). Unless otherwise specified, between-group comparisons used the operated hip as the unit of analysis; GEE models used the patient as the clustering unit to account for correlation between bilateral hips in the same patient. The sample size was determined by the number of eligible cases during the study period, and no *a priori* sample-size or power calculation was performed. After assessment of data distributions, continuous variables were summarized as means ± standard deviations and categorical variables as counts and percentages. Between-group comparisons of continuous variables used the independent-samples *t* test or Welch *t* test, and categorical variables were compared using the chi-square test or Fisher's exact test. Because some expected cell counts were small, the overall distribution of Hartofilakidis types A, B, and C was compared using the Fisher–Freeman–Halton exact test. Reproducibility of continuous radiographic measurements was assessed using ICCs. Interobserver agreement for the main Hartofilakidis classification, individual Moore signs, and binary composite endpoints was evaluated using Cohen's *κ* with 95% CI; the total number of Moore signs was evaluated using quadratically weighted Cohen's *κ*, and observed agreement was reported for signs with no within-sample variability. Complications were reported separately by category as the number and proportion of patients experiencing each event. A patient with events in different categories could be counted in more than one category; therefore, category counts were not summed. Given the small number of events, between-group comparisons of complications were exploratory. All tests were two-sided, and *P* < 0.05 was considered statistically significant.

In addition to *P* values, effect sizes were used to describe between-group differences in the unweighted baseline characteristics: standardized mean differences (SMDs) were reported for continuous variables and Cramer's *V* for categorical variables. To further balance pretreatment characteristics between groups, propensity-score-based stabilized inverse probability of treatment weighting (IPTW) was used as a sensitivity analysis for the primary radiographic endpoint. Propensity scores were estimated using binary logistic regression. Covariates were selected according to pretreatment availability and clinical relevance to implant selection or clinical outcomes rather than on the basis of univariate *P* values. The model included age, sex, BMI, laterality, ASA class, osteoporosis, Crowe classification, consensus Hartofilakidis classification, preoperative CE angle, preoperative Tönnis angle, preoperative H-COR, preoperative V-COR, and calendar time of surgery. Surgery calendar time was continuously encoded from the actual operative date to represent temporal trends during the study period. Variables occurring after implant selection, including screw use, cup size, femoral-head size, high hip center reconstruction, and postoperative COR, were not included in the propensity-score model.

Stabilized weights were defined as *P*(*T* = 1)/PS for the 3D-printed group and *P*(*T* = 0)/(1 − PS) for the conventional group; weights were not truncated. Weighting characteristics were assessed using propensity-score distributions, the common-support range, weight distributions, and effective sample size (ESS). Covariate balance after weighting was assessed using absolute SMDs, with |SMD| < 0.10 considered indicative of good balance. For multicategorical variables, SMDs were calculated for category-specific indicator variables and summarized by the maximum absolute SMD. Covariate balance before and after weighting was displayed using a Love plot. The common-support range was used to assess propensity-score overlap and was not used as a case-exclusion criterion.

Postoperative repeated VAS and HHS measurements were analyzed using Gaussian GEE models with an identity link. Time was treated as a categorical variable, and the models included group, time, and group-by-time interaction terms, with the patient as the clustering unit. A working-independence correlation structure and robust sandwich standard errors were used. Overall longitudinal inference was based primarily on the group-by-time interaction, followed by exploratory time-specific between-group estimated mean differences with 95% CIs and *P* values. Preoperative VAS and HHS values were summarized descriptively and were not included in the postoperative GEE models. To illustrate changes in clinical scores over time, descriptive trajectories of the raw mean ± standard deviation at each time point were plotted for VAS and HHS (Supplementary Figure S3); between-group inference remained based on the GEE models. For the ≥3/5 Moore composite endpoint at 3 months, 6 months, and final follow-up, group, time, and group-by-time interaction effects were analyzed using GEE in the serial-radiograph subset with evaluable images at both 3 and 6 months; time was treated as a categorical variable and the patient as the clustering unit. Unadjusted proportions at each time point were compared using Fisher's exact test. The primary binary radiographic endpoint was compared in the unweighted cohort using Fisher's exact test, with the crude odds ratio (OR) and 95% CI reported. The adjusted analysis used stabilized-IPTW weighted GEE to estimate the between-group effect after balancing pretreatment covariates while avoiding a high-dimensional covariate model for an outcome with a limited number of events. The model specified a binomial distribution with a logit link and used the patient as the clustering unit. Weighted between-group proportions, ORs, 95% CIs, and *P* values were reported. The strict non-RLL endpoint was analyzed using the same approach.

## 3 Results

### 3.1 Patient selection and baseline characteristics

**Figure 2:**
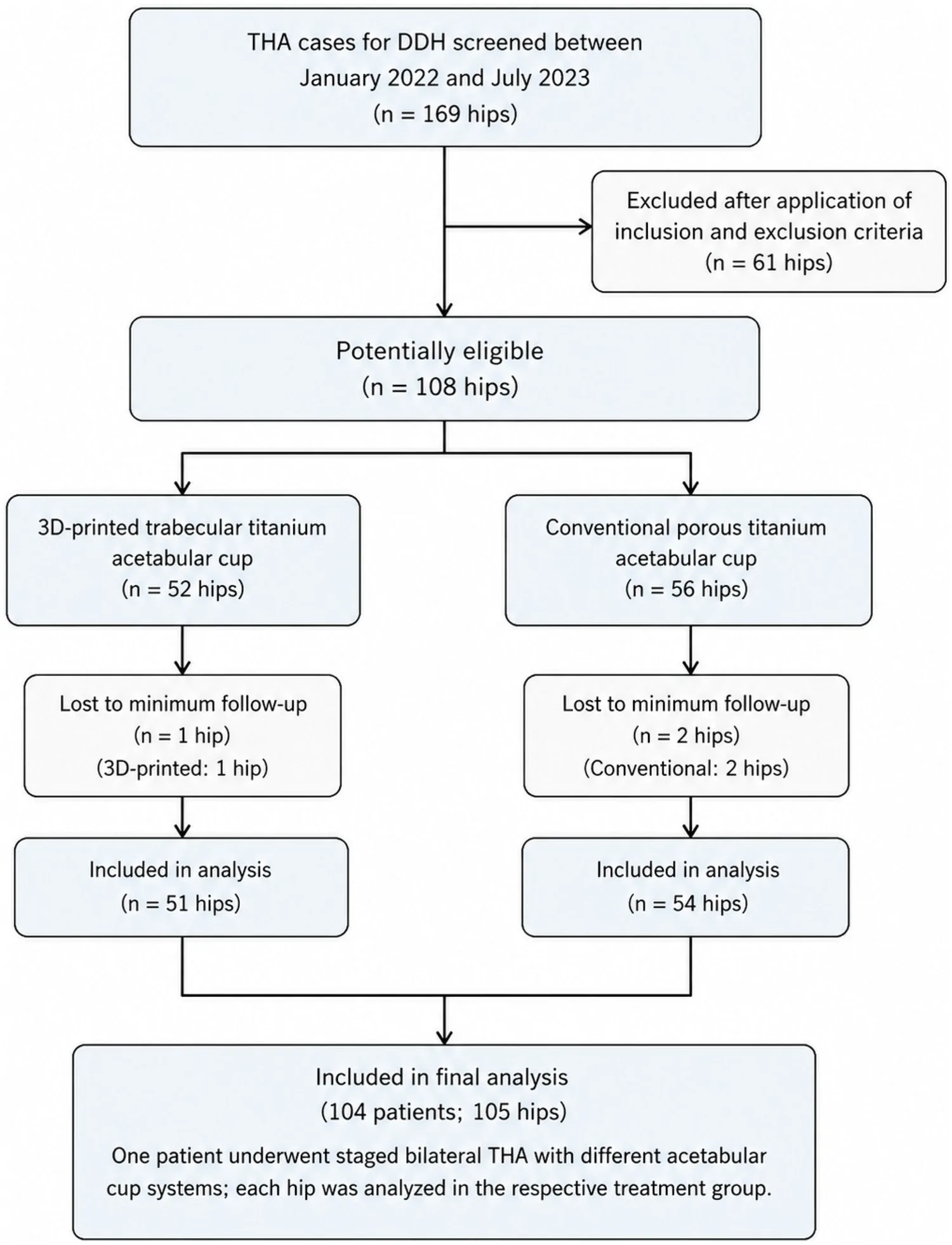
Patient-selection flow diagram

Between January 2022 and July 2023, 169 operative hips undergoing primary THA for DDH were screened. After application of the prespecified inclusion and exclusion criteria, 61 hips were excluded and 108 hips were potentially eligible. Before the minimum follow-up requirement was reached, 3 patients (3 hips) were lost to follow-up, including 1 hip in the 3D-printed cup group and 2 hips in the conventional cup group. The final analysis included 105 hips in 104 patients. Of these, 51 hips were in the 3D-printed trabecular titanium acetabular cup group and 54 hips were in the conventional porous titanium-coated acetabular cup group. One patient underwent staged bilateral THAs, with the two hips assigned to different treatment groups. Mean follow-up was 24.41 ± 5.12 months in the 3D-printed group and 23.91 ± 5.46 months in the conventional group (*P* = 0.626). The patient-selection process is shown in Figure 2.

Patient characteristics, preoperative DDH morphology, surgical period, and implant-related characteristics in the unweighted cohort are shown in Table 1. Observed agreement between the two assessors for the main Hartofilakidis classification was 81.0%, with a Cohen's *κ* of 0.624 (95% CI: 0.477–0.770). After joint review and final consensus classification, types A, B, and C comprised 17 hips (33.3%), 31 hips (60.8%), and 3 hips (5.9%), respectively, in the 3D-printed group and 16 hips (29.6%), 35 hips (64.8%), and 3 hips (5.6%), respectively, in the conventional group. The overall distributions were similar (Fisher–Freeman–Halton exact test, *P* = 0.896; Cramer's *V* = 0.042). Some pretreatment characteristics showed between-group differences in the unweighted cohort, with relatively larger effect sizes for age, ASA class, and Crowe classification; propensity-score weighting was therefore used to further evaluate the primary radiographic outcome.

**Table 1:** Baseline, surgical-period, and implant-related characteristics.

Variable	3D-printed cup group (*n* = 51)	Conventional cup group (*n* = 54)	*P* value	SMD/Cramer's *V*
Patient and preoperative clinical characteristics				
Age, years	57.86 ± 12.52	54.00 ± 12.97	0.124	0.303
Sex			0.300	0.101
Female	32 (62.7%)	39 (72.2%)		
Male	19 (37.3%)	15 (27.8%)		
BMI, kg/m^2^	25.40 ± 3.53	25.46 ± 3.53	0.926	0.018
Laterality			0.938	0.008
Left	24 (47.1%)	25 (46.3%)		
Right	27 (52.9%)	29 (53.7%)		
ASA class			0.274	0.157
I	2 (3.9%)	5 (9.3%)		
II	44 (86.3%)	47 (87.0%)		
III	5 (9.8%)	2 (3.7%)		
Osteoporosis			0.690	0.039
Yes	15 (29.4%)	14 (25.9%)		
No	36 (70.6%)	40 (74.1%)		
Follow-up duration, months	24.41 ± 5.12	23.91 ± 5.46	0.626	0.095
Crowe classification			0.443	0.124
I	23 (45.1%)	18 (33.3%)		
II	16 (31.4%)	22 (40.7%)		
III	12 (23.5%)	14 (25.9%)		
Hartofilakidis classification			0.896	0.042
Type A (acetabular dysplasia)	17 (33.3%)	16 (29.6%)		
Type B (low dislocation)	31 (60.8%)	35 (64.8%)		
Type C (high dislocation)	3 (5.9%)	3 (5.6%)		
Preoperative CE angle, °	9.88 ± 11.91	9.86 ± 11.19	0.993	0.002
Preoperative Tönnis angle/acetabular index, °	49.58 ± 5.46	49.55 ± 4.92	0.976	0.006
Preoperative H-COR, mm	40.29 ± 3.91	40.26 ± 4.31	0.964	0.009
Preoperative V-COR, mm	32.26 ± 11.94	32.24 ± 10.05	0.990	0.003
Surgical-period and implant-related characteristics				
Year of surgery			0.982	0.002
2022	32 (62.7%)	34 (63.0%)		
2023	19 (37.3%)	20 (37.0%)		
Acetabular cup size, mm	53.10 ± 4.51	52.81 ± 3.40	0.718	0.071
Screw use			0.846	0.019
Yes	35 (68.6%)	38 (70.4%)		
No	16 (31.4%)	16 (29.6%)		
Number of screws			0.846	0.019
0	16 (31.4%)	16 (29.6%)		
2	35 (68.6%)	38 (70.4%)		
Liner type			—	0.000
Ceramic	51 (100.0%)	54 (100.0%)		
Highly cross-linked polyethylene (HXLPE)	0 (0.0%)	0 (0.0%)		
Femoral head diameter, mm	33.80 ± 2.57	33.56 ± 1.97	0.581	0.109
Femoral stem type			—	0.000
Cementless biologic femoral stem	51 (100.0%)	54 (100.0%)		
High hip center reconstruction			0.504	0.065
Yes	9 (17.6%)	7 (13.0%)		
No	42 (82.4%)	47 (87.0%)		

All data in Table 1 are from the original unweighted cohort. Standardized mean differences (SMDs) are reported for continuous variables and Cramer's V for categorical variables to describe unweighted between-group differences. Covariate balance before and after propensity-score weighting is shown separately in Supplementary Table S1 and Figure S2. Because some expected cell counts were small, the overall distribution of Hartofilakidis classifications was compared using the Fisher–Freeman–Halton exact test.

Propensity-score distributions showed overall overlap between groups. The propensity-score ranges were 0.155–0.879 in the 3D-printed group and 0.100–0.727 in the conventional group, with an empirical common-support interval of 0.155–0.727; 95/105 hips (90.5%) were within this interval. The maximum stabilized weight was 3.138, and the total effective sample size after weighting was 90.9. After IPTW, the absolute SMD was <0.10 for all pretreatment covariates included in the propensity-score model, with a maximum of 0.081, indicating good balance of measured pretreatment characteristics after weighting (Supplementary Table S1; Figures S1 and S2).

### 3.2 Perioperative outcomes and complications

Operative time (81.39 ± 26.66 min vs. 84.91 ± 25.88 min, *P* = 0.495), intraoperative blood loss (261.96 ± 172.74 mL vs. 247.78 ± 171.46 mL, *P* = 0.674), and length of stay (8.53 ± 2.07 d vs. 9.38 ± 2.56 d, *P* = 0.122) did not differ significantly between groups (Table 2).

**Table 2:** Perioperative outcomes and complications.

Variable	3D-printed cup group (*n* = 51)	Conventional cup group (*n* = 54)	*P* value
Operative time, min	81.39 ± 26.66	84.91 ± 25.88	0.495
Intraoperative blood loss, mL	261.96 ± 172.74	247.78 ± 171.46	0.674
Length of stay, d	8.53 ± 2.07	9.38 ± 2.56	0.122
Wound-healing complication	1 (2.0%)	0 (0.0%)	0.486
Dislocation	3 (5.9%)	0 (0.0%)	0.111
Deep vein thrombosis	5 (9.8%)	1 (1.9%)	0.106
Periprosthetic joint infection	0 (0.0%)	0 (0.0%)	—
Periprosthetic fracture	0 (0.0%)	0 (0.0%)	—
Early aseptic loosening^†^	1 (2.0%)	0 (0.0%)	0.486
Revision during follow-up^†^	1 (2.0%)	0 (0.0%)	0.486

† The case of early aseptic loosening underwent revision and was classified as an early failure event. Complication categories may overlap within the same patient; therefore, the counts were not summed or treated as mutually independent events. An overall patient-level complication rate was not calculated.

Regarding perioperative and follow-up complications, the 3D-printed group had 1 wound-healing complication (2.0%), 3 dislocations (5.9%), 5 DVT events (9.8%), 1 case of early aseptic loosening (2.0%), and 1 revision (2.0%); the conventional group had 1 DVT event (1.9%) and no wound-healing complications, dislocations, early aseptic loosening, or revisions. None of the individual between-group comparisons reached statistical significance. These findings are reported as the number and proportion of patients experiencing each complication category. A single patient could contribute to more than one complication category; therefore, category counts do not represent mutually exclusive patients and were not summed. Given the small number of events, safety findings were interpreted primarily descriptively.

The early revision occurred in a 61-year-old man with right-sided Crowe type III, Hartofilakidis type B DDH and no osteoporosis. The index THA used a 54-mm 3D-printed trabecular titanium acetabular cup without supplemental acetabular screws. Immediate postoperative radiographic measurements showed a cup inclination of 38.1°, anteversion of 13.4°, and a BCI of 86.6%. The operative record indicated subjective primary stability but did not document quantitative insertion torque, interface micromotion, or a standardized press-fit grade. On postoperative day 2, dislocation with acetabular cup migration occurred and revision was performed; supplemental superior screw fixation was used after revision (Figure 5). Because the event occurred before biological bone ingrowth could develop, it was classified as failure of the index cup in the primary analysis.

**Figure 3:**
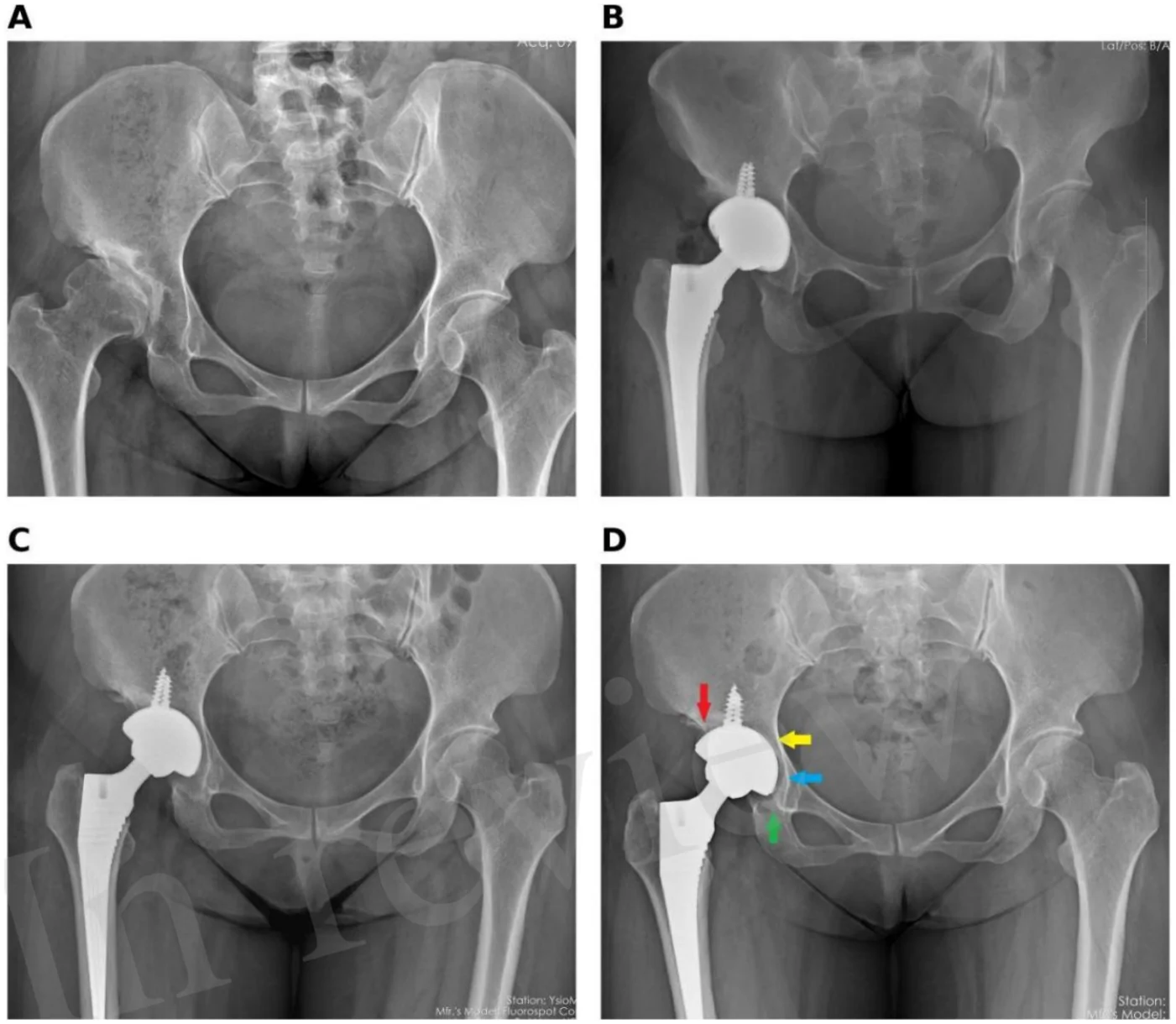
Representative radiographs from a patient in the 3D-printed cup group: **(A)** preoperative anteroposterior pelvic radiograph; **(B)** immediate postoperative radiograph; **(C)** 1-month postoperative radiograph; and **(D)** final-follow-up radiograph. In panel D, the red arrow indicates a superolateral buttress, the yellow arrow indicates absence of a radiolucent line at the cup–bone interface, the blue arrow indicates medial stress shielding, and the green arrow indicates an inferomedial buttress.

**Figure 4:**
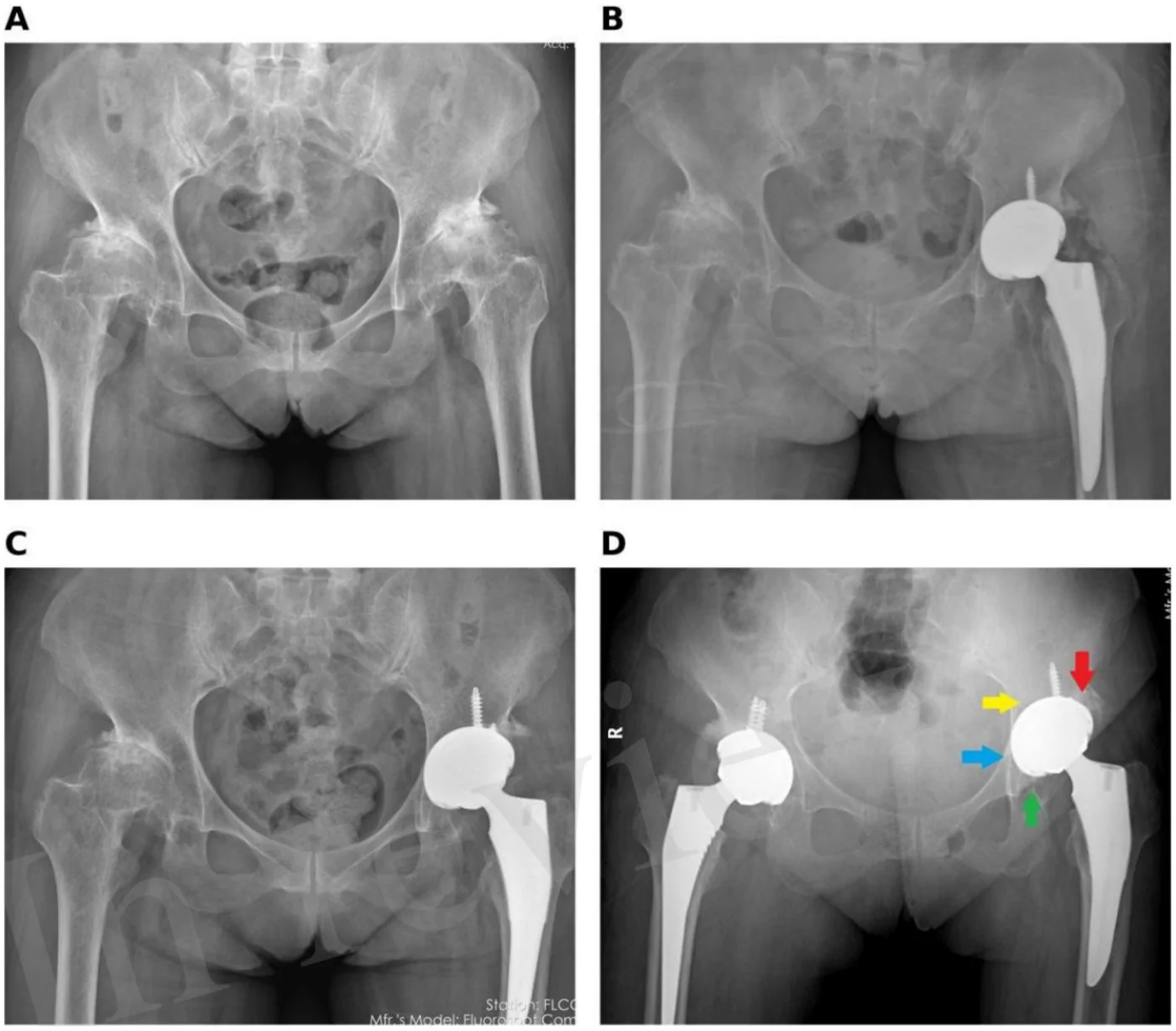
Representative radiographs from a patient in the conventional porous titanium-coated cup group: **(A)** preoperative anteroposterior pelvic radiograph; **(B)** immediate postoperative radiograph; **(C)** 1-month postoperative radiograph; and **(D)** final-follow-up radiograph. In panel D, the red arrow indicates a superolateral buttress, the yellow arrow indicates absence of a radiolucent line at the cup–bone interface, the blue arrow indicates medial stress shielding, and the green arrow indicates an inferomedial buttress.

**Figure 5:**
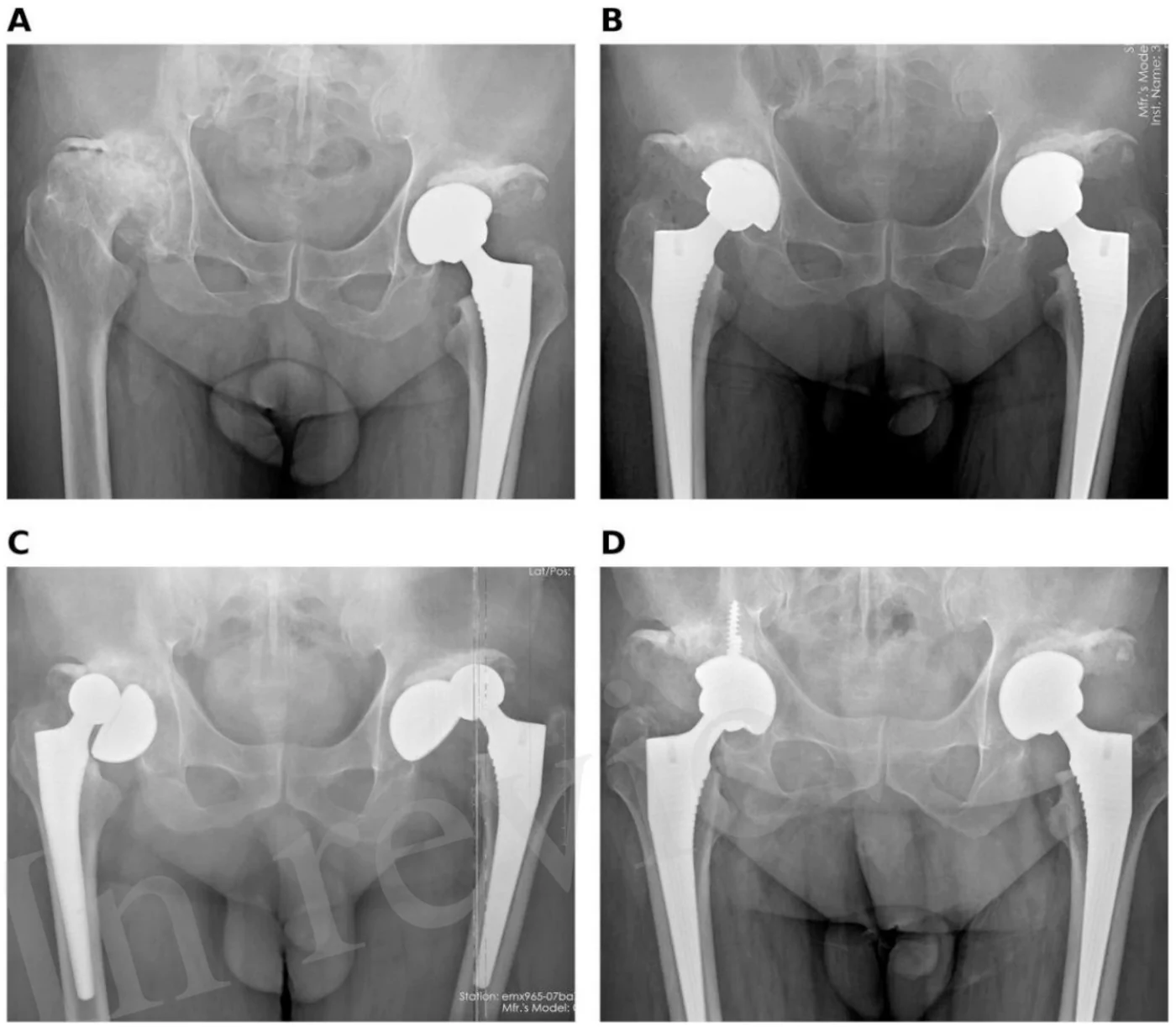
Radiographic sequence of the early aseptic loosening/revision case in the 3D-printed cup group: **(A)** preoperative radiograph; **(B)** immediate radiograph after primary THA, with no evident supplemental acetabular screws; **(C)** postoperative day-2 radiograph showing dislocation and acetabular component migration; and **(D)** post-revision radiograph showing superior screw fixation of the revised acetabular component.

### 3.3 VAS pain scores

Preoperative summary VAS values were similar between groups (4.08 ± 1.38 vs. 4.16 ± 1.43, *P* = 0.771). Preoperative VAS was not included in the postoperative longitudinal GEE model. Postoperative VAS analysis showed a significant time effect (*P* < 0.001) and a group-by-time interaction (*P* = 0.046). The estimated mean difference between the 3D-printed and conventional groups was −0.56 points at 1 week (95% CI: −1.11 to −0.01, *P* = 0.045), −0.55 points at 1 month (95% CI: −1.05 to −0.05, *P* = 0.032), and −0.49 points at 3 months (95% CI: −0.95 to −0.04, *P* = 0.033). At 6 months, the between-group difference was small (−0.03 points, 95% CI: −0.21 to 0.16, *P* = 0.793) (Table 3). Mean VAS trajectories are shown in Supplementary Figure S3A.

**Table 3:** Changes in VAS pain scores over time and longitudinal model results.

Time point	3D-printed cup group (*n* = 51)	Conventional cup group (*n* = 54)	GEE-estimated between-group mean difference, 3D−conventional (95% CI)	*P* value
Preoperative^†^	4.08 ± 1.38	4.16 ± 1.43	—	0.771
1 week	2.88 ± 1.52	3.44 ± 1.37	−0.56 (−1.11 to −0.01)	0.045
1 month	2.43 ± 1.33	2.98 ± 1.32	−0.55 (−1.05 to −0.05)	0.032
3 months	1.76 ± 1.21	2.26 ± 1.18	−0.49 (−0.95 to −0.04)	0.033
6 months	1.22 ± 0.54	1.24 ± 0.43	−0.03 (−0.21 to 0.16)	0.793
Time effect				<0.001
Group × time interaction				0.046

† Preoperative VAS was obtained from baseline summary values and was not included in the postoperative longitudinal GEE model. Postoperative between-group differences are GEE-estimated mean differences (3D-printed group−conventional group). Negative values indicate lower VAS pain scores in the 3D-printed group.

### 3.4 Harris Hip score

Preoperative HHS was similar between groups (45.37 ± 12.51 vs. 46.48 ± 12.31, *P* = 0.648). Preoperative HHS was not included in the postoperative longitudinal GEE model. Postoperative HHS analysis showed a significant time effect (*P* < 0.001) and a group-by-time interaction (*P* < 0.001). The estimated mean difference between the 3D-printed and conventional groups was 3.28 points at 1 month (95% CI: 0.09–6.47, *P* = 0.044), 3.41 points at 3 months (95% CI: −0.30–7.11, *P* = 0.072), and 3.90 points at 6 months (95% CI: 0.67–7.13, *P* = 0.018). At 12 months, 18 months, and final follow-up, the estimated between-group mean differences were 0.01, −0.36, and −0.15 points, respectively, with no statistically significant differences (all *P* > 0.05) (Table 4). Mean HHS trajectories are shown in Supplementary Figure S3B.

**Table 4:** Changes in harris Hip score over time and longitudinal model results.

Time point	3D-printed cup group (*n* = 51)	Conventional cup group (*n* = 54)	GEE-estimated between-group mean difference, 3D−conventional (95% CI)	*P* value
Preoperative^†^	45.37 ± 12.51	46.48 ± 12.31	—	0.648
1 month	79.69 ± 8.11	76.41 ± 8.21	3.28 (0.09–6.47)	0.044
3 months	83.31 ± 10.68	79.91 ± 7.96	3.41 (−0.30–7.11)	0.072
6 months	88.86 ± 7.66	84.96 ± 8.90	3.90 (0.67–7.13)	0.018
12 months	90.45 ± 5.53	90.44 ± 6.92	0.01 (−2.43–2.44)	0.996
18 months	92.51 ± 5.54	92.87 ± 5.45	−0.36 (−2.50–1.78)	0.741
Final follow-up	92.90 ± 7.30	93.06 ± 6.59	−0.15 (−2.84–2.53)	0.911
Time effect				<0.001
Group × time interaction				<0.001

† Preoperative HHS was obtained from baseline summary values and was not included in the postoperative longitudinal GEE model. Postoperative between-group differences are GEE-estimated mean differences (3D-printed group−conventional group). Positive values indicate higher HHS in the 3D-printed group.

### 3.5 Postoperative radiographic reconstruction and acetabular Cup position

Postoperative acetabular cup position and reconstruction parameters were generally comparable between groups. Representative radiographs of a patient in the 3D-printed cup group and a patient in the conventional porous titanium-coated cup group are shown in Figures 3 and 4, respectively. No significant between-group differences were observed in cup inclination (43.12 ± 4.35° vs. 43.09 ± 4.27°, *P* = 0.967), anteversion (15.96 ± 3.14° vs. 15.97 ± 3.18°, *P* = 0.990), Cup-CE angle (17.84 ± 8.06° vs. 17.85 ± 6.27°, *P* = 0.994), BCI (86.13 ± 8.45% vs. 86.16 ± 7.18%, *P* = 0.988), postoperative V-COR (21.47 ± 5.02 mm vs. 21.45 ± 3.45 mm, *P* = 0.979), postoperative H-COR (31.58 ± 4.65 mm vs. 31.56 ± 3.30 mm, *P* = 0.984), the proportion of high hip center reconstruction (17.6% vs. 13.0%, *P* = 0.592), or LLD (3.13 ± 1.74 mm vs. 3.12 ± 1.59 mm, *P* = 0.997) (Table 5).

**Table 5:** Postoperative radiographic reconstruction and acetabular Cup position.

Variable	3D-printed cup group (*n* = 51)	Conventional cup group (*n* = 54)	*P* value
Acetabular cup inclination, °	43.12 ± 4.35	43.09 ± 4.27	0.967
Acetabular cup anteversion, °	15.96 ± 3.14	15.97 ± 3.18	0.990
Cup-CE angle, °	17.84 ± 8.06	17.85 ± 6.27	0.994
Bone coverage index, %	86.13 ± 8.45	86.16 ± 7.18	0.988
Postoperative V-COR, mm	21.47 ± 5.02	21.45 ± 3.45	0.979
Postoperative H-COR, mm	31.58 ± 4.65	31.56 ± 3.30	0.984
High hip center reconstruction	9 (17.6%)	7 (13.0%)	0.592
Leg-length discrepancy (LLD), mm	3.13 ± 1.74	3.12 ± 1.59	0.997

### 3.6 Radiographic signs suggestive of acetabular Cup osseointegration

In the 30-hip reliability sample, observed agreement between the two assessors for the 4 individual Moore signs with variability ranged from 83.3% to 90.0%, with Cohen's *κ* values of 0.638–0.780. Observed agreement for “absence of radiolucent lines” was 100%; *κ* was not estimated because there was no within-sample variation. The quadratic-weighted *κ* for the total number of Moore signs was 0.779 (95% CI: 0.609–0.949), and observed agreement for the ≥3/5 composite endpoint was 96.7%, with a Cohen's *κ* of 0.889 (95% CI: 0.676–1.000) (Supplementary Table S3). For the strict non-RLL endpoint (≥3/4), observed agreement was 76.7%, with a Cohen's *κ* of 0.533 (95% CI: 0.266–0.801) (Supplementary Table S3). Among the 105 hips, 82 (78.1%) had evaluable Moore radiographs at both 3 and 6 months, including 39/51 hips (76.5%) in the 3D-printed group and 43/54 hips (79.6%) in the conventional group; serial-radiograph availability was similar between groups (*P* = 0.814). In this serial-radiograph subset, the proportions meeting the ≥3/5 Moore radiographic criterion in the 3D-printed and conventional groups were 10/39 (25.6%) and 13/43 (30.2%), respectively, at 3 months (*P* = 0.806); 24/39 (61.5%) and 24/43 (55.8%) at 6 months (*P* = 0.657); and 35/39 (89.7%) and 31/43 (72.1%) at final follow-up (*P* = 0.054; Supplementary Table S4). Patient-clustered GEE showed a significant time effect (*P* < 0.001), with a group-by-time interaction of *P* = 0.097. All hips that met the ≥3/5 criterion at 6 months maintained that radiographic status at final follow-up (24/24 in both groups). For analyses of individual Moore signs and the distribution of non-RLL sign counts at final follow-up, the early failure revised on postoperative day 2 was excluded from the relevant denominators because no evaluable final radiograph of the index cup was available; these analyses therefore included 50 hips in the 3D-printed group and 54 hips in the conventional group. No radiolucent lines were observed around any evaluable index cup. The 3D-printed group had higher proportions of superolateral buttress (74.0% vs. 53.7%, *P* = 0.042), medial stress shielding (72.0% vs. 50.0%, *P* = 0.028), and radial trabeculae (82.0% vs. 61.1%, *P* = 0.029); inferomedial buttress was observed in 74.0% and 63.0%, respectively (*P* = 0.293). The overall distribution of non-RLL Moore sign counts was shifted toward higher sign counts (overall *P* = 0.011; Table 6).

**Table 6:** Radiographic signs suggestive of acetabular cup osseointegration according to the moore criteria.

Moore sign/endpoint^†^	3D-printed cup group	Conventional cup group	*P* value
Absence of radiolucent lines	50 (100.0%)	54 (100.0%)	1.000
Superolateral buttress	37 (74.0%)	29 (53.7%)	0.042
Medial stress shielding	36 (72.0%)	27 (50.0%)	0.028
Radial trabeculae	41 (82.0%)	33 (61.1%)	0.029
Inferomedial buttress	37 (74.0%)	34 (63.0%)	0.293
Non-RLL Moore score = 4	20 (40.0%)	8 (14.8%)	0.011^‡^
Non-RLL Moore score = 3	18 (36.0%)	23 (42.6%)	—
Non-RLL Moore score = 2	8 (16.0%)	9 (16.7%)	—
Non-RLL Moore score <2	4 (8.0%)	14 (25.9%)	—
Primary analysis: revision classified as failure	46 (90.2%)	40 (74.1%)	0.042
Sensitivity analysis: revision case excluded	46/50 (92.0%)	40/54 (74.1%)	0.020
Crude OR for the primary endpoint	3.22 (95% CI, 1.07-9.73)	Reference	—
Strict sensitivity analysis: ≥3 of 4 non-RLL signs^¶^	38 (74.5%)	31 (57.4%)	0.099^§^

† Analyses of individual Moore signs and the distribution of non-RLL sign counts included only hips with an evaluable final radiograph of the index cup; because the early revision case had no valid final radiograph of the index cup, the corresponding denominator in the 3D-printed group was 50.

‡ The early revision case was retained and classified as failure of the index cup for both the primary ≥3/5 Moore composite endpoint and the strict ≥3/4 non-RLL composite endpoint.

§ The 4-category distribution of the 4 non-RLL Moore signs was compared using an overall chi-square test.

¶ The strict sensitivity endpoint was compared using Fisher's exact test. The crude OR was calculated from the 2 × 2 contingency table for the primary endpoint.

In the primary analysis, the early revision was classified as failure of the index cup according to the prespecified rule. The ≥3/5 Moore radiographic endpoint was met by 46/51 hips (90.2%) in the 3D-printed group and 40/54 hips (74.1%) in the conventional group (*P* = 0.042; crude OR = 3.22, 95% CI: 1.07–9.73). After exclusion of the early revision case, the corresponding proportions were 46/50 (92.0%) and 40/54 (74.1%), respectively (*P* = 0.020).

Because all index cups with evaluable final-follow-up radiographs showed absence of radiolucent lines, this universally positive item was removed and the remaining 4 Moore signs were analyzed. After exclusion of the early revision case without an evaluable final radiograph of the index cup, the ordered distribution of non-RLL Moore sign counts remained different between groups (*P* = 0.011). In the strict sensitivity analysis based on the 4 non-RLL signs, the early revision remained classified as failure of the index cup; the proportions meeting the ≥3/4 non-RLL endpoint were 38/51 (74.5%) and 31/54 (57.4%), respectively (Fisher's exact test, *P* = 0.099; Table 6).

In the stabilized-IPTW analysis, with the early revision classified as failure of the index cup, the weighted proportions meeting the primary radiographic endpoint were 92.9% in the 3D-printed group and 75.7% in the conventional group, with a weighted OR of 4.21 (95% CI: 1.35–13.17, *P* = 0.013), consistent in direction with the unweighted analysis. For the strict non-RLL endpoint, the weighted proportions were 75.7% and 57.3%, respectively, with a weighted OR of 2.32 (95% CI: 0.96–5.58, *P* = 0.061). Under the stricter radiographic definition, the direction of effect was maintained, although the confidence interval was correspondingly wider (Supplementary Table S2).

## 4 Discussion

The principal finding of this study was that, among patients undergoing primary THA for Crowe type I–III DDH with limited acetabular bone defects not requiring augments or structural bone grafting, use of the 3D-printed trabecular titanium acetabular cup system was associated with a higher proportion of cups meeting the prespecified radiographic criterion at final follow-up than with the conventional porous titanium-coated acetabular cup system. The 3D-printed group also had lower early VAS scores; between-group differences in HHS were observed mainly at selected early time points and converged later in follow-up. Operative time, intraoperative blood loss, length of stay, acetabular cup position, and host-bone coverage were comparable between groups. Complications were infrequent and were interpreted descriptively; one early aseptic loosening with dislocation requiring revision occurred in the 3D-printed group.

This study further characterized preoperative DDH morphology using the Crowe classification, Hartofilakidis classification, and preoperative CE and Tönnis angles. The Crowe classification reflects the degree of proximal femoral-head displacement, whereas the Hartofilakidis classification focuses on the anatomical relationship of the femoral head to the true and false acetabula; the CE and Tönnis angles provide continuous measures of acetabular coverage and acetabular roof inclination, respectively. In the final consensus classification, Hartofilakidis type B accounted for 66/105 hips (62.9%), indicating that the study population predominantly exhibited low dislocation with formation of a false acetabulum. The distribution of Hartofilakidis types was similar between groups and, together with the Crowe classification and continuous radiographic parameters, defined the preoperative morphological context of the study population. From a reconstructive perspective, “limited acetabular bone defects” defined the study population as hips in which primary stability could be achieved with a hemispherical press-fit acetabular cup without a metal augment, reinforcement ring, cage, or structural bone graft. Thus, the study population was characterized at two complementary levels: DDH anatomy and acetabular reconstruction complexity. Interobserver agreement for the main Hartofilakidis classification was 81.0%, with disagreements concentrated mainly at the boundary between types A and B, reflecting whether the femoral head remained within the true acetabulum or had articulated with a formed false acetabulum. Standard anteroposterior pelvic radiographs are suitable for this routine anatomical stratification, whereas standardized CT may provide complementary three-dimensional information regarding the anterior and posterior acetabular walls, local bone stock, and spatial distribution of bone defects.

Safety findings were interpreted by individual complication category. Dislocation and DVT were numerically more frequent in the 3D-printed group than in the conventional group, but the number of events in each category was small, between-group comparisons were not statistically significant, and the two complications have distinct etiologic mechanisms. With respect to dislocation, cup inclination, anteversion, reconstruction of the hip center of rotation, and host-bone coverage were generally similar between groups, and no clear between-group difference was evident from conventional static radiographic parameters. However, postoperative stability in DDH is also influenced by the severity of dysplasia, femoral-head size, femoral version, soft-tissue tension and repair, impingement, and dynamic spinopelvic motion, none of which is fully captured by a static pelvic radiograph. Previous systematic reviews and meta-analyses in patients with DDH likewise indicate that postoperative dislocation is associated with multiple patient- and reconstruction-related factors rather than a single acetabular parameter (25, 59). In a prospective study of 236 primary THAs using the same domestically manufactured 3D ACT acetabular cup, Chen et al. (26) reported 4 in-hospital dislocations (1.7%) and 19 calf DVTs (8.1%), indicating that these events have also been reported in clinical use of a similar implant. With respect to DVT, both groups in the present study followed the same LMWH prophylaxis principles, and operative time, blood loss, and length of stay were comparable. Large contemporary studies suggest that venous thromboembolism after joint arthroplasty is driven more strongly by patient comorbidities and accumulation of multiple risk factors (27). In the unweighted cohort, age and ASA distributions showed some numerical differences in the 3D-printed group, and not all individual thrombotic risk factors were completely recorded; therefore, unmeasured patient-level differences may have contributed to the numerical distribution of DVT events. Accordingly, the numerical differences in dislocation and DVT in this study are more appropriately regarded as exploratory safety observations and should not be attributed to acetabular cup surface architecture.

The observed radiographic differences cannot be attributed solely to the 3D-printing manufacturing process. The two devices differ simultaneously in porosity, pore size, elastic modulus, integrated vs. coated surface architecture, frictional characteristics, and potentially other geometric and manufacturing features. In the present study, additive manufacturing should therefore be regarded as the means of producing a specific porous architecture, whereas the clinical exposure was the complete implant system. Recent materials-science and clinical reviews indicate that there is no consensus regarding the optimal design or manufacturing method for porous acetabular structures (13, 28). General biomechanical, animal, and retrieval studies suggest that surface architecture, pore size, and interface characteristics can influence primary stability and subsequent bone ingrowth (29–31, 58). An animal study of the AK Medical 3D ACT acetabular cup material compared 3D-printed highly porous titanium with plasma-sprayed titanium from the same manufacturer. Bone–implant contact was similar between groups at 45 days, whereas at 90 days the 3D-printed highly porous material showed greater bone–implant contact (32), providing product-related preclinical support for the osseointegration potential of this open porous architecture. These findings complement histologic bone ingrowth observed in retrieval studies of 3D-printed acetabular components (30). At the same time, primary stability and migration may also be affected by host-bone density and local bone quality (33, 34). The radiographic differences observed in this study are therefore more appropriately interpreted as being associated with the overall implant design rather than attributable to additive manufacturing, porosity, elastic modulus, or any other single parameter. Specifically, the more frequent medial stress shielding observed in this study can be regarded as adaptive periacetabular bone remodeling related to redistribution of load (35, 36, 55), whereas radial trabeculae reflect oriented trabecular continuity and remodeling at the cup–bone interface (22). These Moore signs remain indirect findings on plain radiographs and should not be equated with histologically confirmed bone ingrowth or greater fixation strength.

The Moore composite signs integrate multiple plain-radiographic features to assess osseointegration of cementless porous acetabular components and have prior validation (22). We retained the ≥3/5 criterion as the primary radiographic endpoint. Because “absence of radiolucent lines” was universally positive in both groups among evaluable cups, this item was additionally removed in a non-RLL sensitivity analysis. After exclusion of this universally positive sign, the overall distribution of non-RLL Moore sign counts remained shifted toward higher counts. With the stricter binary endpoint of ≥3/4 non-RLL signs, the direction of effect remained consistent, although neither the unweighted nor the IPTW analysis reached statistical significance. This sensitivity analysis indicates that the direction of the primary result was not driven entirely by the RLL item, while also showing reduced precision under the stricter definition.

Serial radiographic analysis showed that the proportion of cups meeting the ≥3/5 Moore criterion increased in both groups from 3 months to 6 months and final follow-up. The between-group proportions were similar at 3 and 6 months, and the group-by-time interaction in the patient-clustered GEE did not reach statistical significance (*P* = 0.097). Thus, the available data did not demonstrate clearly faster early radiographic integration in the 3D-printed group; the between-group difference became more apparent at final follow-up. All hips that met the threshold at 6 months continued to meet the ≥3/5 Moore criterion at final follow-up, suggesting that the composite radiographic signs established by the intermediate follow-up were generally maintained within the study period. The Moore composite signs were originally validated against actual implant fixation status in revision cases, and the presence of ≥3 signs had high predictive value for bone ingrowth (22). In the broader literature on cementless acetabular cups, early fixation behavior may also have long-term prognostic relevance. Previous systematic reviews and meta-analyses have shown that early postoperative cup migration is associated with later revision for aseptic loosening (37). More recently, a study combining radiostereometric analysis data from multiple acetabular cups with long-term outcomes from two national joint registries further demonstrated an association between migration during the first 1–2 postoperative years and the 10-year risk of revision for aseptic loosening (38, 54). These data support the clinical relevance of evaluating early implant fixation and radiographic integration; however, the Moore composite signs themselves have not been established as a surrogate endpoint for long-term implant survivorship. Whether the between-group radiographic differences observed in this study will translate into lower long-term rates of aseptic loosening or revision therefore requires further follow-up.

The 3D-printed group had lower early VAS scores and higher HHS at selected early time points, whereas cup orientation, host-bone coverage, and reconstruction of the hip center were similar between groups. The 1-week postoperative period is more representative of primary mechanical stability than mature osseointegration; therefore, the potential effects of a high-friction, integrated porous interface on cup–bone contact, interface micromotion, and primary stability may provide one explanation for the early differences (29). Both groups followed the same early weight-bearing principles. Lower pain may improve tolerance of early functional rehabilitation, while the HHS itself includes a pain component. Accordingly, the early VAS and HHS differences may reflect the combined effects of the mechanical environment, pain, and rehabilitation tolerance. Actual activity level, rehabilitation dose, and analgesic use were not continuously quantified, and these mechanisms require prospective evaluation.

Clinical studies of 3D-printed acetabular reconstruction in DDH have focused mainly on customized or integrated acetabular reconstruction, and their populations and reconstruction complexity are not fully comparable with those of the present study. Cheng et al. (7) compared a 3D-printed integrated acetabular prosthesis with structural bone grafting in 45 patients with Crowe type II–III DDH. At 3 months, HHS was 87.6 ± 3.1 in the 3D-printed group and 80.2 ± 2.2 in the bone-grafting group; at final follow-up, HHS values were 91.2 ± 3.8 and 90.1 ± 4.6, respectively, with functional outcomes converging over time. Zhao et al. (39) reported 85 patients (106 hips) with Crowe type I–IV DDH treated with a 3D-printed porous integrated acetabular prosthesis; after a mean follow-up of 26.8 months, the final HHS was 90.4 ± 3.3. In the present 3D-printed cup group, HHS was 83.31 ± 10.68 at 3 months and 92.90 ± 7.30 at final follow-up, generally within the range reported in previous studies (52). However, differences in patient composition, implant design, and reconstruction technique preclude direct efficacy comparisons. Unlike previous DDH studies that primarily evaluated integrated prostheses or structural bone grafting, the present study focused on Crowe type I–III DDH that could be reconstructed with a hemispherical press-fit cup without metal augmentation or structural bone grafting, and directly compared an off-the-shelf 3D-printed trabecular titanium cup with a conventional porous titanium-coated cup used during the same period. Contemporary comparative studies of 3D-printed highly porous cups and conventional cups have also begun to emerge in general THA populations (40), but direct comparative evidence in DDH remains limited (56). The present study therefore adds device-level comparative evidence within this specific reconstructive setting (57). The findings apply to the two specific systems and DDH population evaluated here and should not be directly extrapolated to other 3D-printed designs or severe acetabular bone defects.

Both implants were used during the same period, all procedures were performed by the same senior surgeon, and the distribution of surgery year was similar between groups, which reduced temporal and learning-curve effects to some extent. Preoperative acetabular morphology and bone quality, together with intraoperative host-bone coverage and press-fit stability, were used primarily to determine whether the hemispherical press-fit reconstruction strategy evaluated in this study was feasible rather than to assign patients to a specific acetabular cup according to a prespecified rule. When both systems were considered technically suitable, the final choice was made primarily through shared decision-making. Preoperative acetabular morphological parameters, Crowe classification, postoperative cup position, and host-bone coverage were broadly similar between groups, reducing the likelihood that the observed findings were explained by gross morphological differences, although confounding from unmeasured three-dimensional acetabular defects, local bone quality, and intraoperative press-fit quality cannot be completely excluded.

The similar rates of supplemental screw use between groups likewise do not eliminate the potential influence of intraoperative factors, because the decision to add screws depended on the surgeon's assessment of primary stability and host-bone coverage. Biomechanical and long-term clinical studies suggest that screw configuration may influence reconstruction stability, but supplemental screws cannot substitute for adequate host-bone contact and press-fit fixation (41, 42). Three-dimensional planning and simulation studies further show substantial individual variation in acetabular defects, particularly in Crowe type II–III hips, and suggest that preoperative imaging-based planning may help predict augmentation requirements and guide selection of an appropriate reconstruction strategy (43–46).

Acetabular component position determines the postoperative hip center of rotation and thereby influences hip biomechanics, LLD, dynamic joint loading, and the risk of adverse events after THA (47–49). In the present study, postoperative cup inclination, anteversion, Cup-CE angle, BCI, V-COR, H-COR, LLD, and the proportion of high hip center reconstruction did not differ significantly between groups, indicating broadly comparable cup position and bone coverage. This reduces the likelihood that differences in cup position explain the radiographic osseointegration findings, although residual confounding from unmeasured three-dimensional bone defects and local press-fit quality cannot be completely excluded.

One case of early aseptic loosening with dislocation occurred on postoperative day 2 in the 3D-printed group. The patient did not have osteoporosis, and inclination, anteversion, and host-bone coverage of the index cup did not indicate an obvious reconstruction deviation; the operative record also documented subjective primary stability. No supplemental screw was used during the index procedure, whereas supplemental screw fixation was used after revision. Because the event occurred before biological bone ingrowth could develop, its timing is more consistent with early mechanical failure of primary stability. Primary acetabular cup stability is determined by multiple factors, including host-bone contact, actual press-fit, local bone quality, component position, impingement, early loading, and implant surface friction (29, 42). Clinical radiostereometric analysis studies further suggest that supplemental screws are not an independent, singular determinant of cup stability and that reliable press-fit fixation itself can provide favorable early stability (50). Quantitative insertion torque or interface micromotion was not recorded in this study; accordingly, this case is more appropriately interpreted as an early mechanical failure arising from multiple interacting factors. To assess the influence of pretreatment baseline differences on the primary radiographic comparison, stabilized IPTW was additionally performed. After weighting, the absolute SMD for every pretreatment covariate was <0.10, and the direction of effect for the primary conservative endpoint was consistent with the unweighted analysis, indicating that the radiographic association remained stable after balancing measured baseline characteristics. The IPTW result for the strict non-RLL endpoint was also directionally consistent with the unweighted sensitivity analysis.

This study has several limitations. First, it was a single-center retrospective, nonrandomized comparison. Although measured pretreatment characteristics were well balanced after stabilized IPTW, implant selection still involved clinical judgment and shared decision-making; patient preference and cost considerations contributed to the final choice, while related socioeconomic characteristics were not recorded in a structured manner. Local bone quality, actual press-fit quality, and other unmeasured factors may therefore have resulted in residual confounding. Second, the sample size was limited and mean follow-up was approximately 24 months, precluding assessment of long-term differences in aseptic loosening, revision, and implant survivorship between the two cup systems; long-term DDH studies indicate that the risk of implant failure can continue to accumulate beyond 10 years (51). Third, osseointegration was assessed primarily using Moore signs on plain radiographs, and serial radiographs at both 3 and 6 months were available for only 82/105 hips. Fourth, actual activity level, rehabilitation dose, and analgesic use were not continuously quantified, and complication events were infrequent; therefore, comparisons of recovery mechanisms and safety remain exploratory. Finally, the study used an operational definition of “limited acetabular bone defects” and was restricted to Crowe type I–III DDH, so the findings are most applicable to similar hemispherical press-fit reconstruction scenarios.

## 5 Conclusions

In primary THA for Crowe type I–III DDH with limited acetabular bone defects not requiring supplemental reconstruction, the 3D-printed trabecular titanium acetabular cup, compared with a conventional porous titanium-coated acetabular cup, was associated with a higher proportion meeting the final-follow-up Moore radiographic endpoint and with better early VAS and HHS outcomes. The proportions meeting the early Moore radiographic endpoint were similar between groups, and differences in clinical scores converged later in follow-up. These findings pertain to the two specific implant systems evaluated; longer follow-up is required to assess long-term fixation and implant survivorship (53).

## Data Availability

The original contributions presented in the study are included in the article/Supplementary Material, further inquiries can be directed to the corresponding author.
