# Supplementary material for: Short-term clinical and radiographic outcomes of a 3D-printed trabecular titanium acetabular cup in primary total hip arthroplasty for Crowe type I–III developmental dysplasia of the hip with limited acetabular bone defects: a retrospective comparative study
Source: Front Med (Lausanne). 2026 Aug 31;13:1906028. doi: 10.3389/fmed.2026.1906028 (PMC13572131; doi:10.3389/fmed.2026.1906028)
Supplement: Supplementary file 1 [file Datasheet2.doc]

**Figure S1** Propensity-score distributions before weighting. Density curves show the distribution of estimated propensity scores in the 3D-printed and conventional cup groups. Dashed vertical lines indicate the empirical common-support limits (0.155 and 0.727); 95 of 105 hips (90.5%) were within this interval. The common-support interval was used to assess propensity-score overlap and was not used as a trimming criterion.

**Figure S2**. Covariate balance before and after stabilized inverse probability of treatment weighting (IPTW). Absolute standardized mean differences (SMDs) are shown before and after weighting. The dashed vertical line indicates the prespecified balance threshold of |SMD|=0.10. For multicategorical covariates, the plotted value represents the maximum absolute SMD across category-specific indicator variables. After stabilized IPTW, all covariates had |SMD|<0.10, with a maximum value of 0.081.

Figure S3. Descriptive trajectories of VAS and Harris Hip Score (HHS) over follow-up. (A) VAS pain scores. (B) HHS. Data points and error bars represent the raw mean ± SD at each time point. These plots are descriptive; between-group inference was based on patient-clustered Gaussian generalized estimating equation models with a working-independence correlation structure and robust sandwich standard errors. Preoperative VAS and HHS values were presented descriptively and were not included in the postoperative longitudinal GEE models.
